# Methods and Machines in Surgery

**Published:** 1916-10-07

**Authors:** 


### METHODS AND MACHINES IN SURGERY.

Once upon a time, it appears, a surgeon, in an expansive moment, allowed himself to claim that the members of his craft were the most efficient of all hand-workers. There is virtue in such challenges, for they wake up the critics, and the critic, in spite of his rough exterior, is a helpful discipline. Perhaps the event will justify itself in the present instance. Anyhow, the claim of the surgeon has not escaped objection. Everyone has heard of the methods of organisation and of standardisation which have been applied to industries on the other side of the Atlantic, and it is by this standard that the surgical claim is disputed. A Mr. Frank B. Gilbreth, who is said to be the modern apostle of "motion study" as the prime factor of efficiency, has made it his business to study surgical methods as practised at various hospitals, and has come to the conclusion that the surgeon has much to learn from the mechanical industries. His activities, his tools, his surroundings, we are told, all suffer from lack of standardisation. To take first the instruments, the rule is laid down that "great practice with comparatively few tools is one of the laws of the most efficient use of tools." Examined in this light modern surgical procedure is found to be deplorable. So far from cultivating the efficient use of a limited group of instruments, there is a direct incentive to the multiplication of tools. To design something new is a pathway to fame, at least in an instrument-maker's catalogue, and perhaps on a wider platform. Hence the multiplicity of instruments and the limited amount of practice applied to any one of them. The right procedure, so runs the conclusion, is to have a central specially equipped laboratory where every new design may be measured, tested, and compared with existing designs; then in due course will issue the official verdict and fame or condemnation will await the budding inventor. Modern experiences are drilling us in many directions, and "committees" spring into life daily. But we fancy the time is not yet when the surgeon will look for an official benediction on an instrument before deciding to use it. If such a proceeding is advisable, should not the principle be applied all round and the physician be advised what books he should study?

There is probably more to be said for the suggestion that the surgeon is not always fully trained in those methods of manipulation which economise time and effort. His work is largely, at least when operating, mechanical, and, as in other crafts, there are habits and procedures which do not come by nature. What has been done by systematic study of the methods of various workers in numerous industrial occupations is well known to those who have given attention to the subject, and the craft of surgery might well be submitted to a similar investigation. It is not suggested that the surgeon does not usually accomplish the end he intends, but rather that he could do this with a greater economy of time and effort were he trained in the principles of motion-study. It seems that some surgeons who at first resented the proposal have learnt by experience to appreciate its benefits.

